# Prevalence and correlates of cannabis abuse among vulnerable communities following multiple natural disasters

**DOI:** 10.1192/j.eurpsy.2023.818

**Published:** 2023-07-19

**Authors:** G. Obuobi-Donkor, R. Shalaby, E. Eboreime, B. Agyapong, V. I. O. Agyapong

**Affiliations:** 1 Psychiatry, Dalhousie University, Halifax; 2 Psychiatry, University of Alberta, Edmonton, Canada

## Abstract

**Introduction:**

Most individuals use cannabis for relaxation and may misuse this substance. Vulnerable communities who have experienced multiple traumas may be predisposed to cannabis abuse. Hence, more cannabis abuse is deserving of more attention.

**Objectives:**

To determine the prevalence and correlates of likely cannabis abuse among residents of Fort McMurray.

**Methods:**

A cross-sectional survey design was adopted, employing an online questionnaire. Data were analyzed with SPSS version 25. Correlation analysis was conducted to assess likely cannabis abuse and its association with other mental health conditions.

**Results:**

One hundred and eighty-sixed out of the two hundred and forty-nine completed the online survey, giving a response rate of 74.7%. The prevalence of self-reported cannabis abuse was 14%. Most of the participants were females (159, 85.5%), owned their houses (145, 78.0%), and 103 (60.6%) reported being exposed to at least a trauma (COVID-19, flooding, or wildfire). Rented accommodation predicted likely cannabis abuse (OR = 3.86; 95% CI: 1.34–11.14), males were more likely to abuse cannabis than the female gender (OR= 6.25; 95% CI: 1.89–20), and participants in a relationship were more likely to abuse cannabis (OR = 6.33; 95% CI: 1.67–24.39). There was a statistically significant association between depressive and anxiety symptoms and likely cannabis abuse.

**Conclusions:**

The study found an association between depression and anxiety symptoms with cannabis abuse among residents of the Fort McMurray population. Sociodemographic characteristics predispose individuals to problematic cannabis use. Vulnerable communities who have endured multiple disasters need psychological care and support to reduce and prevent cannabis abuse.

**Disclosure of Interest:**

None Declared

